# Expanding application of digital pathology in Japan - from education, telepathology to autodiagnosis

**DOI:** 10.1186/1746-1596-6-S1-S19

**Published:** 2011-03-30

**Authors:** Yasunari Tsuchihashi

**Affiliations:** 1 Louis Pasteur Centre for Medical Research in Kyoto, Kyoto, Japan

## Abstract

**Background:**

Digital pathology, i.e., applications of digital information technologies to pathology practice, has been expanding in the recent decades and the mode of pathology diagnostic practice is changing with enhanced precision. In the present study the changing processes of digital pathology in Japan were investigated and trends to future were discussed.

**Methods:**

The changing status of digital pathology was investigated through reviewing the records of annual meetings of the Japanese Research Society of Telepathology and Pathology Informatics (JRST-PI) and of the Japanese pathology related medical and informatics journals. The results of the Japanese questionnaire survey conducted in 2008-2009 on telepathology and virtual slide were also reviewed. In addition effectiveness of an experimental automatic pathology diagnostic aid system using computer artificial intelligence was investigated by checking its rate of correct diagnosis for given prostate carcinoma digital images.

**Results:**

Telepathology played a central role in the development of digital pathology in Japan. Both macroscopic and microscopic pathology digital images were routinely generated and used for diagnostic purposes in major hospitals. Virtual slide (VS) digital images were used first for education then for conference, consultation and also gradually for routine diagnosis and telepathology. The experimental automatic diagnostic aid system achieved the rate of correct diagnosis around 95% for prostate carcinoma and its use for automatic mapping of cancerous areas in a given tissue image was successful.

**Conclusions:**

Advance in the digital information technologies gave revolutionary impacts on pathology education, conference, consultation, diagnosis, telepathology and also on pathology diagnostic procedures in Japan. The future will be bright for pathologists by the advanced digital pathology but we should pay attention to make the technologies and their effects under our control.

## Background

Digital pathology, i.e., applications of digital information technologies to pathology practice, has been expanding in the recent decades and the mode of pathology diagnostic practice, conference and of education is progressively changing with enhanced efficiency and precision [1-3]. In the present study the changing processes of digital pathology in Japan were investigated through reviewing domestic works and achievements relating to them and trends to future of digital pathology and their problems were discussed.

## Methods

The changing status of digital pathology was investigated through reviewing mainly the records of annual meeting of the Japanese Research Society of Telepathology and Pathology Informatics (JRST-PI) established in 2001 and also reviewing digital pathology related works presented at the Japanese society of pathology, the Japanese society of clinical cytology and at other pathology related medical informatics journals. The reviewed literatures covered the records from the year 1991 to 2009. The results of the recent questionnaire survey on telepathology and virtual slide jointly conducted in 2008-2009 by JRST-PI, Japanese Society of Pathology and by a study group on ‘Clinical Cancer Research’ promoted by the Japan Ministry of Health, Labor, and Welfare were also reviewed. An automatic diagnostic aid system for prostate carcinoma with artificial intelligence was developed in cooperation with DAINIPPON SCREEN MFG. CO., LTD, Japan [9,10]. Pathologist's experience and decision of cancer or non-cancer were taught in advance to the system with studying capabilities and then the system judged a given digital image as cancer or non cancer following the system experience. Digital images of prostate were obtained and they were divided into many unit grid areas of a certain fixed size. Judgment of cancer or not was done by each unit grid basis and the rate of correct diagnosis was obtained. Mapping of carcinomatous areas in a given digital prostate carcinoma image was done using the automatic diagnostic aid system.

## Results

**Figure 1:**
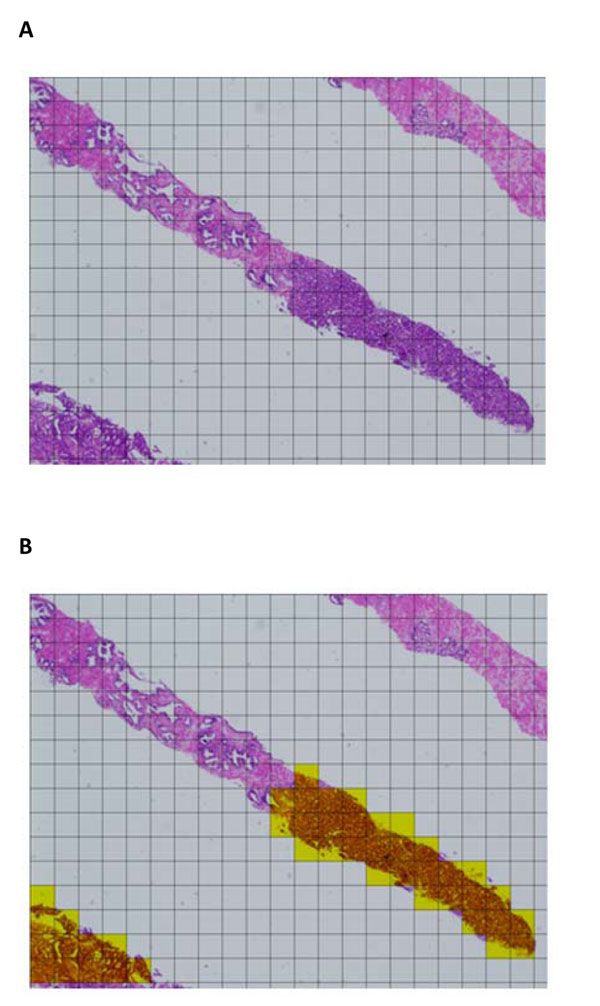
1-A Needle biopsy of prostate carcinoma, HE stained, and the observing field is divided into many unit grid areas for judgment of malignancy or not. 1-B Needle biopsy of prostate carcinoma, HE stained, and the yellowish unit grid areas are judged as containing carcinoma by computer artificial intelligence with studying capability.

A digital public line, ISDN net 64, was first used for a real time, still-image telepathology in Kyoto Japan in 1992 that was just before the prevalence of internet of our country [1]. Since then telepathology has been playing a central role in development of digital pathology. Through prevalence and also through progressing lowering of cost of digital imaging devices, both macroscopic and microscopic pathology images were routinely generated and used for various diagnostic and educational purposes in pathology department of major hospitals [2]. Through the prevalence of virtual slide (VS) systems with its peak introduction year in 2007, when a promotion project for VS by the Japanese government started, utilization of digital images was rapidly expanding and now encompasses fields of education, conferences, consultation, telepathology, and also routine diagnosis [3]. VS digital images were first and most practically used for educational purposes and its trial started in Yamaguchi University in 2004 [4]. In 2006, digitalized pathology images generated by a VS system was incorporated into a part of HIS (Hospital Information System) as PACS (Picture Archiving Communication System) and utilized for diagnosis, conferences and for explanation of disease conditions to a patient in Toyama municipal hospital [5]. Use of VS digital images for telepathology diagnosis was challenged in some institutions [6] and now gradually becoming popular in Japan. Diagnostic consultation of difficult tumour cases using VS images is in progress but at the moment staying in limited use probably due to currently still limited access to VS systems and also for burden on a pathologist of digitalization procedure [7]. Quantification of immunostains using VS digital images was introduced in 2008 and now gradually prevailing in major laboratories. It was centralized somehow and gradually accepted for pathology diagnostic practice on business basis [8]. Use of liquid based cytology (LBC) was a prerequisite for VS digital imaging in cytopathology [9]. In the experimental automatic diagnostic aid system for prostate carcinoma   using computer artificial intelligence the rate of correct detection of cancer unit grid image was at the moment   around 95% and the value was high enough to attain practical mapping of cancer areas of a specimen (Fig.1-A and Fig.1-B). The major developmental steps of digital pathology in Japan were summarized in the Table 1.

**Table 1:** 

Major Developmental Steps of Digital Pathology in Japan	
1991	Open experiment of satellite telepathology challenged (Kyoto)
1992	Use of a digital line, ISDN net 64, for a still image telepathology (Kyoto)
1993	Introduction and prevalence of internet started in academic institutions
1995	Use of digital images in routine surgical pathology started (Kyoto)
1997	Web based internet telecytology started (Hokkaido)
2003	Virtual slide (VS) system development, domestic in Japan (Aomori)
2004	Use of VS system for pathology laboratory education (Yamaguchi)
2005	Network conferences using VS systems challenged
2006	Use of VS images in hospital information system (Toyama)
2007	VS telepathology using a public optic fibre line started (Kyoto)
2007	Introduction of VS system supported by Japanese government having its peak
2008	Quantification of immunostain VS images introduced
2009	Quantification of immunostain VS images on business (Toyama)
2009	Diagnostic consultation of difficult tumour cases using VS in National Cancer Centre, Tokyo, in progress (Tokyo)
2009	Diagnostic aid system development challenged (Kanagawa)
2009	Experimental diagnostic aid system for prostate carcinoma (Kyoto)

## Discussions

Utilization of digital information for pathology practice started as a shift from analogue to digital in telepathology that included all the basic elements of digital pathology i.e., image capture, processing, storing and transfer [1]. Telepathology has been a leading modality in the development of digital pathology and also a mother of virtual slide (VS) or whole slide imaging (WSI) development. Now virtual slide (VS) images or whole slide imaging (WSI) are playing a central role in digital pathology. VS images are currently most practically used for educational and conferencing purposes [3] mainly due to their mass effects and to their liberation from the issue of protection of patient privacy. Utilization of VS images in hospital information system (HIS) is theoretically very useful not only for a pathologist but also for clinicians, co-medical staffs, patients and also for patient's family [5]. But its practical use requires further lowering of costs of memory devices and also full staffs that enable regular and stable running of the imaging system routinely generating digital pathology images. Enabling automatic and reliable generation of VS images that are tolerable for routine pathology diagnosis is still a continuing technical issue. For consultation of difficult pathology cases VS images will be more and more frequently used in near future [7]. But the basic issues that how can we make good traffic control for an authority diagnosis or how we can establish reasonable decision making in consensus diagnosis on a net are still matters of debate. One front of development of digital pathology is a field of autodiagnosis. By utilizing digital image technologies and computer artificial intelligence a part of diagnostic works of a pathologist will be done automatically. In the present study the automatic diagnostic aid system for prostate carcinoma gave 95% correct detection of cancerous areas and succeeded in automatic mapping of carcinomatous areas in a given digital image. The use of the system will be a great help for pathologist works, however appropriate division and sharing of man and machine works and also pathologist's and computer's works will be important. Future will be bright for pathologists by the advanced digital pathology but we should pay attention to make always the technologies and their effects under our control.

## Conclusions

The digital pathology was in steady progress in Japan. It has been lead by telepathology and its applications were ever expanding in the recent decade. VS imaging now has a pivotal position in the digital pathology and is currently most frequently used for educational, conferencing and consultation but its use for routine pathology diagnosis and also for telepathology diagnosis are in gradual progress. Development of autoanalytic/diagnostic systems using computer artificial intelligence are frontier of digital pathology and will change drastically the basic mode of pathology practice in future.

## Competing interests

The authors declare that they have no competing interests.
